# Living with epilepsy in Lubumbashi (Democratic Republic of Congo): epidemiology, risk factors and treatment gap

**DOI:** 10.11604/pamj.2015.21.303.5580

**Published:** 2015-08-26

**Authors:** Béatrice Koba Bora, Didier Malamba Lez, Daniel Okitundu Luwa, Marcellin Bugeme Baguma, Désiré Tshala Katumbay, Tharcisse Kayembe Kalula, Pierre Luabeya Mesu'a Kabwa

**Affiliations:** 1 Department of Neurology, School of Medicine, University of Lubumbashi, Democratic Republic of Congo; 2 Centre Neuro-Psychiatrique Joseph Guislain, Lubumbashi, Democratic Republic of Congo; 3 Department of Internal Medicine, School of Medicine, University of Lubumbashi, Democratic Republic of Congo; 4 Centre Neuro-Psycho-Pathologique, School of Medicine, University of Kinshasa, Democratic Republic of Congo; 5 Department of Neurology, Oregon Health & Science University, Portland, Oregon, United States of America; 6 Oregon Institute of Occupational Health Sciences, Oregon Health & Science University, Portland, Oregon, United States of America; 7 Centre Psychiatrique Saint-Martin Fracarita Dave, Belgium

**Keywords:** Epilepsy, prevalence, Congo, Treatment gap, Traditional healers, conception of epilepsy

## Abstract

**Introduction:**

Epilepsy is the most common of serious neurological disorders, yet despite considerable efforts, good access to medication, appropriate social and societal acceptance and acceptable quality of life (QoL) are difficult to achieve especially in developing countries. It is estimated that over 500,000 people suffer from epilepsy in the DRC. There is no report, in our knowledge on the epilepsy in Lubumbashi.

**Methods:**

A descriptive study was undertaken in individuals with a clinical diagnosis of epilepsy who presented at the CNPJG outpatient clinic in Lubumbashi over a 12 months period. A 64-item questionnaire was used to collect information from the patients. Case records were reviewed and relevant demographic, social, professional, medical history, medical condition data were extracted.

**Results:**

Among 3,540 patients who presented to a neuropsychiatric clinic run by the Fracarita charity over a 1-year period, 423 (11.9%) were identified as having epilepsy, and 179 were subsequently included in the survey after they (or their parent/guardian) provided informed consent and completed an EEG investigation. Data were collected using a standardized, 64-item questionnaire. Epilepsy had negative impact on the lives of individuals with the condition; 40.8% had either no education or had completed primary education only, 38.0% were unemployed and the majority (64.6%; n = 113) were unmarried or divorced. Family history of epilepsy (first or second degree) was present in 23.5% of cases. Other reported factors that could potentially precipitate epilepsy included obstetric and perinatal factors (15.1%) and central nervous system infections during infancy (8.4%). Consumption of alcohol or recreational drugs accounted for 10.6%. The treatment gap was above 67% and the delay between first seizure and first consultation was 15 months. When asked to describe their condition, or its cause, 55.3% of participants (or their families) considered epilepsy to be of spiritual/ religious origin, while 25.1% had almost no insight and could not provide any description.

**Conclusion:**

This first epidemiological study shows a high prevalence of epilepsy among patients presenting to the clinic in Lubumbashi, DRC, and reveals a significant treatment gap.

## Introduction

Epilepsy is the most common of serious neurological disorders, yet despite considerable efforts, good access to medication, appropriate social and societal acceptance and acceptable quality of life (QoL) are difficult to achieve. Attaining these goals in developing countries, where approximately 80% of people with epilepsy (PwE) live [1], presents even greater challenges. The prevalence of epilepsy in sub-Saharan Africa and Latin America is particularly high; 15.0 and 17.8 per 1,000 people, respectively, compared with 6 per 1,000 in Asia and lower to 8 per 1,000 in Europe and North America [2]. The first, and to our knowledge, only epidemiological data on epilepsy from the Democratic Republic of Congo (DRC) were published in 1970 [3]. Of 4,400 people seen at the Neurology Department of the University Hospital of Lovanium in Kinshasa, 865 were identified as having epilepsy (19.7%). A preliminary analysis of 300 patients demonstrated that more than half experienced generalised convulsions. The same team published data on 1,356 consultations at the paediatric neurology department in the period between January 1975 and October 1976 with a total of 310 convulsions observed in children up to 6 years of age [4]. Lubumbashi is the capital of the southern province of Katanga, and with a population of over 1.5 million, it is the second most important city in the DRC, after the capital, Kinshasa. It is the major commercial and industrial hub of the country, given it rich mineral resources. In 2007, the Centre Neuro-Psychiatrique Joseph Guislain (CNPJG) was established in the city by the Brothers of Charity (Fracarita Belgium). The charity is primarily active in the fields of mental health, care for people with a disability and education and training in 32 countries worldwide. Since 2010, healthcare workers at CNPJG have been specifically focusing on epilepsy, not only in treating patients with the conditions, but also raising awareness through educational programmes. In this epidemiological study we sought to document several elements; the number of patients presenting to the neuropsychiatric clinic of brothers of charity with epilepsy and their characteristics, potential risk and aetiological factors for epilepsy, delay to diagnosis and access to anti-epileptic treatment.

## Methods

A descriptive study was undertaken in individuals with a clinical diagnosis of epilepsy who presented at the CNPJG outpatient clinic in Lubumbashi over a 12 months period, from May 2010 to April 2011. Patients were deemed eligible for analysis if they were over 6 years old and if they agreed to undergo electroencephalography (EEG). When possible, a CT scan has been realised. It was only for people who have financial possibilities with a clinical status indicate this exam. All patients provided informed consent before inclusion in the study. A 64-item questionnaire was used to document demographic data, and other information such as education level, societal status, religion, medical history, characteristics of epileptic seizures, time to diagnosis, current and previous treatment, including traditional healing, and access to anti-epileptic drugs (AEDs). Data were collected by the treating physician. Case records were reviewed and relevant demographic, social, professional, medical history, medical condition data were extracted. Each patient underwent general physical and neurological examination and eligible patients had realised an EEG. Demographic and epilepsy-related data were analysed using descriptive statistics (Epi Info^™^ version 3.5.1 (CDC, Atlanta, USA)). Continuous variables were summarized as mean ± standard deviation if normally distributed. Median was used for non-normally distributed variables. Categorical variables were summarized as proportions and frequencies. The Chi square and KRUSKAL-WALLIS test were used to compare differences between groups. Treatment gap was defined as the difference between the number of people with active epilepsy and the number of those receiving appropriate treatment at the time of the study, expressed as a percentage.

## Results

During the 12-month observation period, 3,540 patients presented at the CNPJG with different neuro-psychiatric conditions of whom 423 (11.9%) were identified as having epilepsy; 239 (56.4%) were male and 184 (43.6%) female. Of the person living with epilepsy, 179 were included in the survey after they provided informed consent and completed an EEG investigation. The mean age of the study population was 21.5 years, range 6 - 106 years (Table 1). Whereas 88% lived in Lubumbashi, only 55.3% of the patients were originally from the province of Katanga. A total of 40.8% had either no education or had completed primary education only and 38.0% were unemployed. Of 113 patients who were questioned about marital status 75 (64.6%) were unmarried, divorced or single. A family history of epilepsy (first or second degree) was present in 23, 5% of cases, suggesting a genetic pathology in some patients (Table 2). Epilepsy was observed more frequently among first-born children (57%), than in second- and third-born (14.0% and 16.2%, respectively). Other factors that could have potentially contributed to the development of epilepsy were obstetric and perinatal factors in 15.1% of patients, followed by central nervous system infections during infancy (8.4%). Of interest is the consumption of alcohol or recreational drugs such as cannabis accounting for 10.6%. Over 60% of patients could not recall a precipitating factor for their condition. When asked to describe their condition, 12.8% provided a good description - i.e., kifwafwa (tonic-clonic seizures), ndeke (crisis with foam to the lips), musanfu (febrile convulsions in children).

**Table 1:** Demographic characteristics of study participants

Demographic characteristics	N = 179
**Age [years, mean (range)]**	21.5 (6 - 106)
**Gender male / female [number (%)]**	
Male	101 (56.4)
Female	78 (43.6)
**Education [number (%)]**	
No	29 (16.2)
Primary school	44 (24.6)
Secondary school	83 (46.4)
High school or university	23 (12.8)
**Province of origin [number (%)]**	
Katanga	99 (55.3)
Kasai	60 (33.5)
Kivu	10 (5.6)
Bandundu	3 (1.7)
Equator	1 (0.6)
Bas-Congo	2 (1.1)
Others	4 (2.2)
**Marital status [number (%)] of 113 PwE**	
Married (total /male / female)	38(33.6) / 19 (16.8) / 19 (16.8)
Single (total /male / female)	73 (64.6) / 46 (40.7) / 27 (23.9)
**Occupation [number (%)]**	
Without employment	68 (38.0)
Student	71 (39.7)
Craftsman	11 (6.1)
Self-employed	17 (9.5)
Trade/business	12 (6.7)
**Religion [number (%)]**	
Catholic	71 (39.7)
Protestant	85 (47.5)
Other	23 (12.8)

**Table 2:** Medical history and types of seizure experienced by study participants

Clinical parameters	N = 179
Medical history	**n (%)**
Family history of epilepsy	42 (23.5)
Alcohol consumption	16 (8.9)
Meningo-encephalitis	15 (8.4)
Perinatal suffering	14 (7.8)
Prematurity	13 (7.3)
Others (neonatal icterus, Sickle-cell disease)	9 (5.0)
Head injury	6 (3.4)
Presence of a neurological condition	4 (2.2)
Use of recreational drugs	3 (1.7)
Not known	57 (31.8)
Seizure type	
Tonic-clonic generalised	103 (57.5)
Partial complex	17 (9.5)
Partial secondary generalised	16 (8.9)
Partial simple	14 (7.8)
Variable crisis	12 (6.7)
Atonic crisis	10 (5.6)
Absences	6 (3.4)
Hyperkinetic crisis	1 (0.6)
Precipitating factors	
Emotions	15 (8.4)
Ending AED treatment	12 (6.7)
Others (fever, strong odors, etc)	12 (6.7)
Menses	8 (4.5)
During sleep	8 (4.5)
Alcohol intoxication	6 (3.4)
Use of recreational drugs	4 (2.2)
After sleep deprivation	3 (1.7)
After awakening	2 (1.1)
Not known	109 (60.9)

In contrast, 55.3% of PwE considered their condition to be of spiritual and/or religious origin. An additional 6.7% of patients confused malaria with epilepsy. One out of four patients (25.1%) could not provide any description. Generalised tonic-clonic seizures were the most frequently reported type of seizure (57.5%), while the other clinical presentations of epilepsy all remained below 10%. The EEG was taken within 72 hours after the last seizure, with 66.5% showing abnormalities (Table 3). In total, 171 abnormalities were counted in 119 patients with an abnormal EEG. Abnormal slow waves were noted in 51 patients, while fast waves were noted in only 5 patients. Focal slow waves and diffuse slow waves were observed in 44 and 31 patients, respectively. Focal spikes (or sharp waves) were seen in 32 patients, whereas diffuse or generalised spikes were seen in 7 patients and 1 patient, respectively. Thirty-seven patients have undergone a CT scan. In 59.5% no abnormalities were noted. Among the remaining patients with abnormal scans, ischemic cerebrovascular accidents were the most frequent observation (18.9%). At the time of the first presentation at the CNPJG, only 33% of participants had received AEDs (Table 4) indicating a treatment gap of 67%. Of the remaining patients, 30% had received no treatment at all, 20% had received incorrect treatment (e.g., antimalarials, antibiotics), while 16.8% had received traditional treatment. Both religion and province didn't contribute to treatment choice. A total of 179 patients received treatment at the centre. The vast majority of patients (92.2%) received monotherapy, with valproate being the most commonly used AED (48.6%), followed by carbamazepine (23.5%) and levetiracetam (17.3%). Most patients continued their treatment (79.9%) and most achieved good seizure control (82.0%). The most frequently reported treatment-emergent adverse events were somnolence (62%), slowing of motor and cognitive function (48%) and ataxia (18%).

**Table 3:** Results of neurological investigations

EEG parameters	
Waves	**n (%)**
Normal	60 (33.5)
Abnormal	119 (66.5)
Wave pattern	
Abnormal slow waves	51
Abnormal fast waves	5
Focal slow waves	44
Diffuse slow waves	31
Focal spikes	32
Diffuse spikes	7
Generalised spikes	1
CT-scan	37 (100)
Ischemic cerebrovascular accident	7 (18.9)
Cerebral atrophy	2 (5.4)
Multifocal hyperdensities	2 (5.4)
Syndrome of Fahr	1 (2.7)
Astrocytoma	1 (2.7)
Tuberculoma	1 (2.7)
Sinusitis	1 (2.7)
Normal	22 (59.5)

**Table 4:** Treatment-related parameters

Treatment	Number (%)
Treatment received prior to first consultation	179
AED	59 (33.0)
None	54 (30.2)
Incorrect, *e.g*., antimalarias, antibiotics	36 (20.0)
Traditional	30 (16.8)
Traditional treatment by religion	29
Catholicism	9 (12.7)
Protestantism, Pentecostalism	20 (23.5)
Others	0 (0)
Traditional treatment by province	28
Katanga	21 (21.1)
Kasai	7 (11.7)
Treatment received at CNPJG	179
Monotreatment	165 (92.2)
*Valproate*	*87 (48.6)*
*Carbamazepine*	*42 (23.5)*
*Levetiracetam*	*31 (17.3)*
*Phenobarbital*	*4 (2.2)*
*Phenytoin*	*1 (0.6)*
Combination treatment	14 (7.8)
Continuation of treatment received at CNPJG	179
Yes	143 (79.9)
No	36 (20.1)
Adverse events of AED treatment	
Somnolence	62
Slower motor and cognitive capacity	48
Ataxia	18
Hyperactivity	5
Others, *e.g*., vertigo, fatigue, weight gain	24

## Discussion

Epilepsy is the most common neurological condition worldwide, however, prevalence and incidence data are often lacking in low-income countries. Only historical epidemiological data are available for epilepsy in the DRC [3, 4]. Results from our study at the CNPJG in Lubumbashi, established the prevalence of epilepsy among a population of patients with neurological disorders as 11.9%. This result should not be confounded with prevalence data in the general population in Sub-Saharan Africa, which varies significantly from study to study, ranging from 2.91 to 14 per 1000 [5–8]. Such variability could arise from numerous factors, including differences in definitions; nature of epilepsy studied (lifetime vs. active); sample size; timing and method of sampling and the sample itself, *i.e*, children *vs*. Adults; general population *vs*. Population attending specialised centers; rural vs. urban population; clinical *vs*. technically confirmed epilepsy; qualification of healthcare professionals involved in the study and finally prevalence of infectious diseases or other conditions with epilepsy-related sequelae, such as brain trauma or stroke [2, 6, 7, 9, 10]. Epilepsy has a profound impact on the quality of life of individuals affected and their family. Many affected children and adolescents are excluded from schooling, as illustrated in our population with over 40% having received no education or only finishing primary education levels. Also, many PwE and parents caring for the children with epilepsy have limited financial resources to seek adequate care or diagnosis and to purchase AEDs. The negative impact of epilepsy on the ability of individuals to form long-lasting relationships was also shown in our study; among 113 individuals who were questioned about their marital status, the majority (64.6%) reported being unmarried or divorced. A clear description of patients’ condition was provided by the individual patient in only 12.8% of cases, which is in line with the findings of a study conducted in a tertiary health facility in south western Nigeria, where only 10.9% of patients had any insight into the aetiology of their condition [11]. Lack of adequate knowledge about their condition render PwE vulnerable to myths, stigma and exclusion from their social and family networks [12]. Over 60% of participants considered their condition to have a spiritual or religious origin or confounded their condition with malaria. Importantly, 25.1% ignored the presence of a neurological condition altogether.

The development of epilepsy is strongly associated with adverse perinatal and obstetric factors. In our study, 15% of participants reported an abnormal delivery or prematurity, in line with published data [5, 13]. Perinatal and obstetric problems are important risk factors for developing epilepsy in low-income countries. Maternal hygiene and nutrition, obstetric practices and location of delivery (home vs healthcare center) are among the important factors influencing the outcome of the delivery [2]. It is of interest to note that epilepsy was observed more frequently in first-born children. This high prevalence of epilepsy among the firstborn could be explained by the lack of experience of young mothers in recognition of the evolution of a parturition leading to births at home or in vehicles during the transfer to a maternity in precarious conditions and thus traumatic for the newborn with probably head injuries as a cause for epilepsy. Other studies have also reported respectively 7.1% and 20% head injuries as cause of epilepsy [13, 14]. Alcohol consumption as cause of epilepsy was lowers (9%) than reported in literature, for example 12% reported in southeast Nigeria [14]. In our study, we report an epilepsy treatment gap of at least 67% in the city of Lubumbashi, based on our observation that only 33% of participants had received AEDs at the time of their first presentation at the CNPJG. Furthermore, the dosage, dosing schedule and indication of the AEDs were not always appropriate and adjustments were needed. The 67% treatment and access gap is in line with the reported 75% of PwE living in low-income countries being untreated [15–17]. Over 30% of study participants had at some stage been seen by traditional healers and had received traditional herbal medicine (*i.e*., roots, barks and leaves of trees and plants) or were treated with rites and prayers. This is consistent with findings from Northern Tanzania [18] and Cameroon [19]. In Cameroon, it was documented that PwE consider healers to have a good understanding of epilepsy and their attitude toward PwE is deemed satisfactory. Participants’ religion and province of origin did not influence their decision to receive traditional treatment. In terms of religion, protestants and pentecostalists were more likely than catholics to use traditional medicine (23.5% and 12.7%, respectively), while 21.1% of participants from Katanga compared with 11.7% from Kasai used traditional medicine but there was no statistically significant difference(P = 0,08 and 0,126). In our study, an average gap of 15 months between the first visit to the traditional healer and the neurologist at the CNPJG was found. Of the 179 participants in our study, 82% achieved good seizure control. The majority of treated patients (79.9%) continued their AED therapy. Reasons for discontinuation included poverty, negligence, spiritual or religious belief and adverse events. The most frequently reported AED-related adverse events were somnolence and slowing of motor and cognitive function. Further research is required to evaluate the use of AED treatment in Africa, including potential metabolic interactions with concurrent infectious diseases or drug. This could potentially have a negative impact on therapeutic efficacy of the drugs with direct consequences for public health [20, 21].

## Conclusion

Despite the limitations of the study, our results indicate the high prevalence of epilepsy in Lubumbashi and the important treatment gap. The latter is most likely to be higher in rural areas around the provincial capital, as access to adequate and continuous healthcare, as well as access and adherence to treatment are more limited. Our results also highlight the need to reduce the time interval between the first visit to a traditional healer and the first consultation at a specialised centre. Significant efforts are currently under way to reduce this delay in diagnosis and subsequent treatment. Well directed health educational programmes about the causes of epilepsy and the treatment of PwE can play an important role in improving the perception of epilepsy.
